# Supplementary material for: Evaluation of the added value of Brain Natriuretic Peptide to a validated mortality risk-prediction model in older people using a standardised international clinical assessment tool
Source: PLoS One. 2022 Nov 18;17(11):e0277850. doi: 10.1371/journal.pone.0277850 (PMC9674136; doi:10.1371/journal.pone.0277850)
Supplement: Attachment — Submitted filename: PONE-D-22-10241 Response to Reviewers.docx [file pone.0277850.s001.docx]

PONE-D-22-10241 Response to Reviewers

Evaluation of the added value of Brain Natriuretic Peptide to a validated mortality risk-prediction model in older people using a standardised international clinical assessment tool

*Thank you Dr Nickel for your considered review. We have responded under each point below.*

Reviewers' comments:

Reviewer's Responses to Questions

**Comments to the Author**

1. Is the manuscript technically sound, and do the data support the conclusions?

The manuscript must describe a technically sound piece of scientific research with data that supports the conclusions. Experiments must have been conducted rigorously, with appropriate controls, replication, and sample sizes. The conclusions must be drawn appropriately based on the data presented.

Reviewer #1: Yes

*Response: Thank you.*

2. Has the statistical analysis been performed appropriately and rigorously?

Reviewer #1: I Don't Know

*Response: As the first author, and an experienced biostatistican, I appreciate the honesty of this response.*

3. Have the authors made all data underlying the findings in their manuscript fully available?

The [PLOS Data policy](https://apc01.safelinks.protection.outlook.com/?url=http%3A%2F%2Fwww.plosone.org%2Fstatic%2Fpolicies.action%23sharing&data=05%7C01%7Cjohn.pickering%40otago.ac.nz%7Cf8a282542ab94156758c08da4a2b681d%7C0225efc578fe4928b1579ef24809e9ba%7C0%7C0%7C637903847732234051%7CUnknown%7CTWFpbGZsb3d8eyJWIjoiMC4wLjAwMDAiLCJQIjoiV2luMzIiLCJBTiI6Ik1haWwiLCJXVCI6Mn0%3D%7C3000%7C%7C%7C&sdata=mycQVNG9fI6YnnP0%2BlLvNxWzN%2FqfEWwa8pJmnNJ6DH0%3D&reserved=0) requires authors to make all data underlying the findings described in their manuscript fully available without restriction, with rare exception (please refer to the Data Availability Statement in the manuscript PDF file). The data should be provided as part of the manuscript or its supporting information, or deposited to a public repository. For example, in addition to summary statistics, the data points behind means, medians and variance measures should be available. If there are restrictions on publicly sharing data—e.g. participant privacy or use of data from a third party—those must be specified.

Reviewer #1: No

*Response: Because of restrictions of use of clinical data we cannot make it freely available. However, we can make it available if contacted subject to ethics approval from a New Zealand ethics board and a formal use agreement between ourselves and those who wish to use the data. For New Zealand data this is particularly important because some of the data is from Māori, the indigenous people of New Zealand, who are acknowledged to have sovereignty of their data and its use.*

4. Is the manuscript presented in an intelligible fashion and written in standard English?

PLOS ONE does not copyedit accepted manuscripts, so the language in submitted articles must be clear, correct, and unambiguous. Any typographical or grammatical errors should be corrected at revision, so please note any specific errors here.

Reviewer #1: Yes

*Response: Thank you.*

5. Review Comments to the Author

Please use the space provided to explain your answers to the questions above. You may also include additional comments for the author, including concerns about dual publication, research ethics, or publication ethics. (Please upload your review as an attachment if it exceeds 20,000 characters)

Reviewer #1: Evaluation of the added value of Brain Natriuretic Peptide to a validated mortality risk prediction model in older people using a standardised international clinical assessment tool

The authors explore the question if addition of the cardiac biomarker BNP adds prognostic value to a established mortality prediction score in elderly adults in NZ.
This is a well-designed study with a well-phenotyped cohort and fair sample size. Improving risk prediction in the elderly (mean age >80 in this cohort) is of crucial clinical importance for the patient, patient’s family and physician. The main findings of this study are (1) adding BNP to the RiskOP model significantly improved the accuracy of one-year mortality prediction, (2) especially in patients without a diagnosis of CHF. The study utilizes advanced statistical models.

Major
BNP or NT-proBNP is a valuable prognostic biomarker in a wide range of cardio-vascular diseases. BNP is influenced by age, BMI, inflammation, gender, atrial fibrillation and kidney function. The authors should adjust for those metrics before performing survival analyses.
The authors should explain how BNP levels were incorporated in the RiskOP Model. Were age appropriate thresholds used?

*Response: Age, Sex, and BMI are part of the RiskOP model (see end of the 1^st^ paragraph of the methods section) , therefore from a statistical analysis perspective it is not appropriate to account for them a second time (i.e. their influence, through RiskOp, is already accounted for). Unfortunately, we do not have information on AF and kidney function. We have acknowledged this limitation in the discussion:*

*“Additionally, which the RiskOP model incorporates Age, Sex, and Body Mass Index, other factors which may be associated with BNP such as inflammation, kidney function, and atrial fibrillation were not measured or included as covariates. If this was able to be done these may well improve the accuracy of the risk prediction.“*

*BNP concentrations were included as a continuous variable. Dichotomising at any threshold would effectively throw away information and make the models poorer. We have added the phrase “(as a continuous variable)” in the description of the model at the end of the second paragraph of the statistical analysis section.*

It would be helpful for the reader to report the actual BNP values of the cohorts.
The authors should explain why they chose BNP as a biomarker, why not serum sodium, albumin or CRP?

*Response: Thank you for the suggestion. We have added the information on BNP concentrations to the first paragraph of the Results. We chose BNP simply because it is one of the most frequently measured biomarkers in Heart Failure patients in New Zealand. We thought this adequate to assess the proof of concept that the addition of a biomarker to risk prediction from a standardised assessment can improve mortality prediction (line 347). Future studies may look at multi-biomarker response and we have now introduced this possibility into the discussion, we had already indicated that sodium or albumin may be included, but have now added CRP and creatinine.*

The mean age was >80 years old, individuals at this age have a very high change for diastolic dysfunction. Do the authors have access to echocardiograms in the CHF cohort? Is there a way to report the prevalence of Afib in both cohorts?

*Response: Unfortunately, we do not have access to echocardiograms or AF information. We note that in this cohort of people with high-needs, in New Zealand echo-cardiograms are often not done. We have acknowledged the limitation in the limitations section (as in the addition we made in response to an earlier point raised by the referee).*

The authors should clarify when how the RiskOP model was derived, is this part of the interRAI assessment?

*Response: The development of the RiskOP model was published separately with detail on how in Lancet eClinical Medicine (reference 11). It would be too much detail to include here. We have clarified in the text that it was derived on people with a Home Care interRAI assessment (interRAI-HC) – i.e. the same as all people in this present study. The sentence in the introduction now reads:*

*“*The authors recently developed and validated a mortality risk model (RiskOP) for those people aged 65 plus using data from the interRAI-HC only[11].”

And we added a sentence in the methods:

“The RiskOP model had been developed in a subset of these patients (up to March 2018)[11].”

Are the authors suggesting that BNP should be part of a routine assessment in the elderly? Could they estimate a benefit-cost ratio?

*Response: No, we are not suggesting it should be part of a routine assessment in all elderly. Any conclusion we draw must be limited to the high-needs cohort (in this case identified by needing an interRAI Home Care assessment). What we are demonstrating in this proof-of-concept is that a biomarker, in this case BNP, could make risk prediction for mortality better. It is too early to suggest routine adoption, the consequences and benefits of better risk prediction need to be discussed by the medical community. Additionally, we need to assess the added value of other biomarkers, such as creatinine and CRP, which could be measured at the same time. We believe only then a cost-benefit analysis would be applicable (we cannot estimate with on the current data). We have expanded the discussion around future directions:*

*“Further research would need to be undertaken in order to ascertain the value of including additional commonly used blood tests for the older adult population such as sodium, creatinine, albumin, and C-reactive protein. This research would not only need to ascertain the statistical improvement in risk prediction, but also look at a cost-benefit analysis, and ideally would include calibration of models for specific groups including in New Zealand Māori and Pacific Peoples.”*

Image quality of the figures needs to be improved

*Response: Thank you – probably a product of the pdf generation, we will work with the editors to ensure good quality.*

Minor

*Response: Thank you. Unfortunately, these line and page numbers do not correspond with the ones on our documents, nevertheless, we have tracked down these as best we can.*

page 9, 69, sentence needs improved syntax

*Response: We were uncertain on the exact sentence referred to, but revised the entire page and found a sentence with a syntax issue which we fixed.*

page 11, 102, doe the authors mean “informed” instead of “inform”?

*Response: the sentence is “...to make informed decisions...” and is therefore correct.*

page 12, 146, what do the authors mean by “cost effective”?

*Response: Simply that its costs are reasonable compared to other costs (ie it is inexpensive). We have rephrased this to avoid confusion.*

page 17, 236, this is confusing. So how many BNP measurements were included in the analysis?

*Response: Just one per participant, we have added the word “single” in the methods to clarify and changed the wording of the sentence in question.*

Page 17, table 1 the column Died should have the figure 1 deleted

*Response: Thank you, deleted.*

Page 19, 258 should read “died” not “die”

*Response: Thank you, corrected.*

Page 19, 268, Fig2, should read “died” not “dies”

*Response: Thank you, corrected.*

Page 20, 283 and 284, should read congestive heart failure

*Response: Oops – thank you (I must now check the work I’m doing at the same time with CAD that I’ve not made the opposite error there!).*

Page 20, 290, sentence needs improved syntax

*Response: We have reworded.*

6. PLOS authors have the option to publish the peer review history of their article ([what does this mean?](https://apc01.safelinks.protection.outlook.com/?url=https%3A%2F%2Fjournals.plos.org%2Fplosone%2Fs%2Feditorial-and-peer-review-process%23loc-peer-review-history&data=05%7C01%7Cjohn.pickering%40otago.ac.nz%7Cf8a282542ab94156758c08da4a2b681d%7C0225efc578fe4928b1579ef24809e9ba%7C0%7C0%7C637903847732234051%7CUnknown%7CTWFpbGZsb3d8eyJWIjoiMC4wLjAwMDAiLCJQIjoiV2luMzIiLCJBTiI6Ik1haWwiLCJXVCI6Mn0%3D%7C3000%7C%7C%7C&sdata=1EpmJgbRZH2F4JhJg6lZX%2FjJSPlV6mgk40lLmd2sKPs%3D&reserved=0)). If published, this will include your full peer review and any attached files.


If you choose “no”, your identity will remain anonymous but your review may still be made public.


**Do you want your identity to be public for this peer review?** For information about this choice, including consent withdrawal, please see our [Privacy Policy](https://apc01.safelinks.protection.outlook.com/?url=https%3A%2F%2Fwww.plos.org%2Fprivacy-policy&data=05%7C01%7Cjohn.pickering%40otago.ac.nz%7Cf8a282542ab94156758c08da4a2b681d%7C0225efc578fe4928b1579ef24809e9ba%7C0%7C0%7C637903847732234051%7CUnknown%7CTWFpbGZsb3d8eyJWIjoiMC4wLjAwMDAiLCJQIjoiV2luMzIiLCJBTiI6Ik1haWwiLCJXVCI6Mn0%3D%7C3000%7C%7C%7C&sdata=jWort2vY14yXrl%2Brg26j0AKxNwav0pPgfhJYGTKkAf0%3D&reserved=0).

Reviewer #1: **Yes:** Nils Patrick Nickel
