# Supplementary material for: Evaluation of the added value of Brain Natriuretic Peptide to a validated mortality risk-prediction model in older people using a standardised international clinical assessment tool
Source: PLoS One. 2022 Nov 18;17(11):e0277850. doi: 10.1371/journal.pone.0277850 (PMC9674136; doi:10.1371/journal.pone.0277850)
Supplement: Attachment — Submitted filename: Response to Reviewers R2 220927.docx [file pone.0277850.s002.docx]

Dear Editor and Reviewers,

Thank you for the opportunity to respond and the positive reaction to the manuscript. Reviewer 2 has made a few comments, particularly with respect to wanting some additional graphs and comments to help medical people not so familiar with some recent statistical practices better understand the manuscript. We are happy to make the suggested addition. Full response to reviewer’s comments below.

Regards

John Pickering

27 Sept. 2022.

Reviewers' comments:

Reviewer's Responses to Questions

**Comments to the Author**

6. Review Comments to the Author

Please use the space provided to explain your answers to the questions above. You may also include additional comments for the author, including concerns about dual publication, research ethics, or publication ethics. (Please upload your review as an attachment if it exceeds 20,000 characters)

Reviewer #1: Excellent paper! Very interesting for the readership. The authors did an excellent job in answering the reviewers questions.

*Thank you.*

Reviewer #2: 1) If the authors have adequately addressed your comments raised in a previous round of review …

I had the opportunity to read the questions of the first reviewer and the answers of the authors. To my knowledge, they answered correctly to the remarks. The manuscript was adapted correctly.
2) Is the manuscript technically sound, and do the data support the conclusions?

Absolutely.
3) Has the statistical analysis been performed appropriately and rigorously?

Appropriately: this article might be interesting for clinicians. But no single clinician understands more than 10% of the statistics applied. The question is, if so many analyses are necessary for this relatively simple question. "μηδέν άγαν" (meden agan)! Never too much, said Socrates. So why all these analyses, while a simple ROC says enough in this case. Moreover, the promised basic ROC curve (graph) is missing.

Rigorously: decision curve analysis: crucial is here the balance between giving erroneously a bad message, or erroneously a good message. The authors boldly put this balance at 1/1, which is against the philosophy of medical decision making. The key task in decision analysis is always the estimation of this balance. Without a thorough even qualitative research concerning this balance, decision curve analysis makes no sense.

*I love the Socrates quote – after >150 publications I think this is a first for me. Regarding the question, why all these analyses, the answer is simply that a ROC curve is not enough. The lead author is a scientist and experienced biostatistician. I try and use best practice when assessing the added value of biomarkers, but appreciate that not all is readily understandable by people in other disciplines (also Statistics is a young discipline and best practice is changing quickly). Statisticians recognise that ROC plots and AUC alone, are inadequate, hence the other measures. This is because ROC plots and AUC are based merely on ranking, and do not use the full data available when considering biomarkers or prediction models as providing more information. The other issue with ROC plots is that while they have become popular, they are also regularly misinterpreted. It is common to interpret the diagonal on the ROC plot as representing binary chance (coin toss), rather than just one point on the plot (Sensitivity = 0.5, 1- Specificity = 0.5) – I’ve recently published on this [Carrington AM, Fieguth PW, Mayr F, James ND, Holzinger A, Pickering JW, et al. The ROC Diagonal is not Layperson’s Chance: a New Baseline Shows the Useful Area. Machine Learning and Knowledge Extraction. Vienna, Austria: Springer; 2022. page 100–13]. Having said that, I am willing to add ROC plots (they were not promised, merely they were acknowledged as the source of the AUC), to the manuscript.*

*Concerning decision curve analysis and decision making. We certainly take the referee’s point and have added to the limitations section. We absolutely agree that for the implementation of any medical decision making algorithm the net-benefit / harm of actions taken (or not) at any decision threshold must be taken into account. These could differ for the same algorithm at decision thresholds used to rule-out a disease/treatment option or to rule one in or risk stratify to a high-risk. i.e. at one threshold the ratio of benefit to harm may be quite different than at another. This is well beyond the scope of this paper for, as we have tried to emphasise in the text, this is a proof of concept study and so we cannot go further. The test added is:*

“Finally, the net benefit and decision curve analysis is merely a tool for understanding if BNP could be of added value in decision making. For it to be seen as suitable for clinical practice, a more comprehensive net-benefit analysis would be needed weighing both benefits and harms at each decision point, e.g. for risk stratifying patients to low, intermediate, or high risk.”

5) Is the manuscript presented in an intelligible fashion and written in standard English

The English is perfect. But I think even some statisticians will have difficulty understanding all analyses done. I had to go back to the difficult decision curve analysis, to understand fully what the authors mean.
As the authors state that this article might be interesting for clinicians, I propose to show the basic ROC curves with AUC statistics. And to state clearly the difference between significance and effect size. The latter is very small in this research, even in non-cardiac patients. The authors use the word ‘marginally’ in the first paragraph of the discussion, but go further with ‘clear added value’.

*Please see the response concerning ROC curves in our response to question 3.*

*I think the referee is talking about clinical significance rather than statistical significance. Just in case it is the latter: there has been no use of the concept of statistical significance in this manuscript as it is well past its use-by-date. It is recognised as being based on an arbitrary (no biological or clinical meaning) threshold that has resulted in biasing the literature. The likes of the American Statistics Association recommend not using it [Wasserstein RL, Lazar NA. The ASA’s Statement on p-Values: Context, Process, and Purpose. The American Statistician. 2016;70:129–33.]. If it is the former, the difference is that clinical significance is a judgment based on the effect size. The phrase “clear added value” does not appear in the manuscript, so we are a little confused by this. However, we did use the phrase “real value” in the first paragraph of the discussion which referred specifically to the sub-group without CHF. This was not defined, so we replaced “real value” with the term “additional prognostic value.” We simplified the sentence to read.*

“More specifically, the study indicated it had additional prognostic information for clinicians when treating those patients without a diagnosis of CHF and with a risk prediction of below 0.75 (<75% probability of dying within 1-year).”

6) Abstract
Methods: the authors state: “Incremental value was assessed by change in Area Under the Receiver Operating Characteristic Curve (AUC)”
In the results section we (clinicians) miss a classical ROC curve. Instead, we see graphs that take time to understand.

*Please see the response concerning ROC curves in our response to question 3.*

Conclusions: I did not understand immediately the conclusions. The authors conclude that the BNP improves the prediction for non-cardiac patients. I think I’m not the only reader to expect BNP to influence prediction especially in cardiac patients. I would state this as an unexpected paradox: BNP has an added value only in non-CHF.

*Perhaps “paradox” is too strong a word. Mortality is driven by many things which the comprehensive RiskOP model has taken into account. That BNP alone is a predictor of mortality in CHF patients is not part of the study, merely that, in this cohort of people with high needs, it adds no incremental value to those other factors in the RiskOp model. This fact is the purpose of doing studies. We have acknowledged that BNP not adding to the RiskOP model is surprising with the addition of a couple of sentences in the discussion:*

“That there is little additional prognostic value of BNP in those with heart failure may seem surprising. This could simply be that as the people with CHF are more likely to die the covariates in the RiskOP model better correlate with BNP meaning it has less affect on overall mortality prediction. While intriguing, further investigation is beyond the scope of this manuscript.”

Suggestion:
After revision by the first reviewer, I should state this is an excellent article, but not for clinicians or Plos One. In the present writing, I would submit it to Medical Decision Making, given the thorough statistical analyses (except for the decision curve analysis, which should be revised). If it is intended for Plos One, I would revise it along the suggestions I made hereabove.

*We chose PlosOne deliberately because of its policy being focussed on good science rather than utility. Its intended audience is not simply clinicians, but also the biochemists, and biostatisticians working in the field and those engaging with the interRAI instruments and data. As a proof of concept, it is a bit early for a journal like “Medical Decision Making.”*

Reviewer #3: In this manuscript, Pickering et al. performed a proof-of-concept study to evaluate the potential of adding BNP to the one-year mortality prediction model. The study included >1500 individuals with completed interrail-HC assessment and BNP measurement. It was found that the addition of BNP would increase AUC by 0.015 (95% CI 0.004-0.028). The improvement mostly came from individuals without CHF. Overall, the manuscript is well-written.

Following are some minor comments.
1. In the abstract, please define CHF. Given that more than half of individuals had CHF, such information could be also included in the abstract.

*Done, thankyou*

2. Please also include the number of mortalities in the abstract

*Done, thankyou*

3. The sample size used for the calculation is unclear. In Table 2, it is 1537, but in the Results section, it is indicated that 1572 individuals had all the required data. The total number of individuals with CHF (n=785) and without CHF (n=752) would be 1537. This number should be also used in the abstract, but not 1690 individuals.

*We agree it is confusing. The numbers were this way because they reflected the process of finding all with BNP measured within 90d of the interRAI assessment and then removing those with insufficient data for the subsequent calculations. We have simplified to only use the 1537 number in the abstract, refer the 1690 in the results, but only report on the demographics of the 1537 (Table 1).*

4. It would be useful to comment on why the addition of BNP only improved the prediction performance for individuals without CHF but not for those with CHF.

*This indeed is intriguing. However, it is also beyond the scope of the paper as a proof of concept. We have added the following to the discussion:*

“That there is little additional prognostic value of BNP in those with heart failure may seem surprising. This could simply be that as the people with CHF are more likely to die the covariates in the RiskOP model better correlate with BNP meaning it has less affect on overall mortality prediction. While intriguing, further investigation is beyond the scope of this manuscript.”

5. Is there any sex-specific difference in the prediction performance?

*Sex has been taken into account because it is one of the variables in the RiskOp model. To specifically tease out differences in predictive performance by sex would lengthen the manuscript considerably. As the principal purpose is to demonstrate that a measured biomarker (in this case BNP) can be added to interRAI data to add value to a prediction model, we believe that further investigation of differential performance in sub-groups could wait until contemporaneous biomarker measures are made at the time of the interRAI assessment. We have discussed this in the Limitations section and added the sentence:*

*“*Further investigation would also need to assess performance in sex, age, and ethnicity sub-groups.*”*
